# Digital Health Interventions Providing Behavioral Assessment and Goal Prioritization Support: Scoping Review

**DOI:** 10.2196/68112

**Published:** 2025-08-28

**Authors:** Ilona Margaret McNeill, Ron Borland, Charles Abraham

**Affiliations:** 1 Department of Psychological Sciences School of Health Sciences Swinburne University of Technology Hawthorn Australia; 2 School of Psychology Deakin University Geelong Australia

**Keywords:** self-assessment, digital coach, health self-management, goal prioritization, personalized support, mHealth interventions, digital health intervention, DHIs, behavioral assessment, scoping review, systematic review, affordability, health care system, health behavior, behavior change, behavioral change, behavior patterns

## Abstract

**Background:**

Affordability of health care systems depends on populations’ engagement in preventive health behavior and appropriate self-management of long-term conditions. Digital health interventions (DHIs) could facilitate this by prompting and supporting individual health behavior change. Behavior change is often undermined by suboptimal prioritization of goals. Therefore, DHIs aiming to promote behavior change should help users identify behavior patterns that need changing and scaffold goal prioritization.

**Objective:**

This scoping review explores the extent to which DHIs are supporting users to identify and prioritize goals relevant to managing and improving health.

**Methods:**

The review followed the PRISMA-ScR (Preferred Reporting Items for Systematic Reviews and Meta-Analyses extension for Scoping Reviews) guidelines. Web of Science (Core Collection), Scopus, Ovid (Embase, MEDLINE, PsycINFO, and Global Health), and EBSCOHOST (Academic Search Complete and CINAHL Complete) were searched for literature on the development and evaluation of digital interventions that (1) assess users’ current health or health-related behaviors and (2) offer support on prioritization of health-related goals.

**Results:**

Fifty-six papers were included. These identified 19 unique DHIs. Targeted populations included the general population (n=10), those at risk of or diagnosed with cardiovascular disease (n=4), those at risk of or diagnosed with diabetes (n=2), those diagnosed with cancer (n=2), or those diagnosed with HIV (n=1). One DHI targeted preconception among African American women. All DHIs targeted physical activity and most (n=17) targeted diet and smoking, closely followed by alcohol use (n=15) and mental health (n=13). Social wellbeing (n=5), sleep (n=4), and pain (n=1) were less commonly included. All 19 DHIs included a health risk assessment with feedback identifying health domains in need of improvement, but only four asked users to select a prioritized change goal. Outcome evaluations were conducted for most (n=14), with nine DHIs evaluated using at least one randomized control trial (RCT). Almost half of all DHIs (n=9) reported at least one evaluation of behavioral outcomes, mostly employing RCTs (7/9). Six of 19 reported at least one evaluation of psychological health outcomes, again mostly employing RCTs (5/6). Among the seven DHIs for which behavioral outcomes were evaluated using a RCT, effects were mixed, with only one DHI showing significant effects across all assessed behavioral outcomes. Three found significant effects for some, but not all, outcomes or timepoints, and three found no significant effects.

**Conclusions:**

Although all 19 DHIs provided some advice about which health-related goals to prioritize, most did not actively prompt users to set such priorities. DHIs showing the most promise in terms of health behavior change were those that explicitly promoted users to prioritize changing specified health behaviors. This review highlights how DHIs could provide greater behavior change support and provides the basis for designing more effective DHIs.

## Introduction

### Digital Health Resources for Better Health Self-Management

The availability of digital health (or eHealth) resources that aim to facilitate health and well-being has grown exponentially. Estimates of available mobile health applications range from 54,000 to more than 350,000 [1], with health and fitness apps alone said to have yielded US$4.45 billion in revenue in 2023 [2]. This does not include the abundance of websites offering health support. Following this growth, research has expanded rapidly with a focus on the impact of such resources on health behavior change [3]. Researchers have used several terms to refer to technology-based resources that aim to change behaviors, including persuasive technology [4], persuasive systems [5], and behavior-change support systems, which Oinas-Kukkonen et al [6] defined as, “a sociotechnical information system with psychological and behavioral outcomes designed to form, alter, or reinforce attitudes, behaviors or an act of complying without using coercion or deception” (p. 1225). We will use the term “digital health interventions” (DHIs) to refer to such systems.

The convergence of digital technology and health care has generated accessible DHIs that have the potential to promote individual health self-care across geographic, chronological, and socioeconomic boundaries. Previous reviews of such DHIs and other digital health promotion resources have been conducted and have highlighted and discussed variability in intervention content [7-9], but have failed to identify key intervention components that could distinguish between more or less effective DHIs [10]. Identifying these elements is crucial given that the results of DHI evaluations have been less than promising. For example, a meta-analysis of 156 “rigorous” health app evaluations [11] found that they conferred “a slight or weak advantage over standard care” (p. 10) with high heterogeneity of findings, meaning some delivered positive effects while others did not. So why is the substantial effort devoted to DHI development resulting in such limited health impact? We focus on the importance of goal selection and goal prioritization.

### The Need for Identification and Prioritization of Goals

One reason for limited impact may be that DHIs start in the wrong place, with many DHIs assuming that the user has already prioritized goals advocated by the DHI [12]. In reality, users may be considering such goals in the context of multiple valued goals and without much thought regarding prioritization [13-16]. This lack of prioritization increases the chances of unsuccessful goal pursuit.

Goal prioritization is critical because people have limited cognitive and emotional resources to devote to goal pursuit [17-19]. The presence of multiple valued goals competing for such resources increases the likelihood of people shifting their focus away from any particular goal towards another, resulting in poor goal persistence and failure to establish routines and habits that can sustain new behavior patterns over time [20-24]. Goal prioritization has been shown to support sustained goal pursuit in the face of competing goals by providing protection against goal shifting [25-28]. This includes health goal prioritization, which has been shown to lead to greater health behavior change [25,26].

Whilst some users will have already learnt to deploy goal prioritization, many will not. Engaging and helping this latter group with prioritization, particularly those with multiple health needs and at high risk of poor health outcomes, is fundamental to community and population-level health improvement [29]. Such support should result in better individual choices in terms of health goal pursuit and greater commitment to goals. This, in turn, will increase the chance of successful change and, thereby, optimize population change in relation to future health and well-being.

Some DHIs provide goal prioritization support through inclusion of health risk assessments (HRAs). HRAs invite users to reflect on and report their health and health behavior patterns using questionnaires and sometimes laboratory (eg, blood) tests. This is generally followed by personalized feedback identifying assessed health domains (eg, physical health, diet, and sleep) or specific behavior patterns (eg, for diet: consumption of saturated and trans-fats) that can reduce health risks. HRAs were widely used to promote health behavior change in the 1980s [30,31], and digital HRAs are still widely available through worksites, universities, health care organizations, insurance companies, and governmental health department websites. Although HRAs can collect health behavior data and provide feedback using low-cost and accessible tools, their effectiveness as standalone interventions has been limited [32,33]. A newer generation of HRAs, which also assess psychological antecedents of behavior change, such as motivation, readiness to change, perceived need to change, and confidence in change success (ie, self-efficacy) [34], have shown improved effectiveness [35,36]. Nonetheless, there is considerable variability in design and effectiveness of digital HRAs and it is unclear what constitutes best design practice.

### Objective

Research indicates that DHIs will be more effective if they provide support for goal prioritization. We suggest that DHIs should begin by assessing a comprehensive range of health indicators and health-related behavior patterns and, where possible, additional indices (such as biometrics and health diagnoses). Such assessment would enable the identification of health goals that would have the greatest impact on a user’s health. A DHI could then provide practical advice on goal selection and prioritization based on the likely costs and benefits from both an epidemiological perspective (ie, potential health gains) and the user’s situation and preferences, including their abilities and readiness to pursue specific goals. Once priorities had been established, such DHIs could provide behavior change support for prioritized goals, including techniques shown to promote behavior change, such as defining SMART goals and implementation intentions (or If-Then planning) [37,38].

The aim of this review was to identify the availability of DHIs that provide support for health-related goal identification and prioritization. We sought to identify DHIs that include two key elements of behavior-change support. First, an assessment of the user’s current health status or health behaviors across at least two health domains (eg, physical activity and sleep) to enable identification of priority domains or behavior patterns in need of change. Second, some form of support for goal prioritization. We characterize *how* assessment and prioritization support were operationalized in DHIs and describe evaluation practices and findings.

## Methods

### Data Sources and Search Strategy

We developed the protocol for this scoping review following the guidelines of the PRISMA-ScR (Preferred Reporting Items for Systematic Reviews and Meta-Analyses extension for Scoping Reviews; Multimedia Appendix 1) [39]. No additional measures were taken to mitigate potential selection or publication biases. The protocol was extensively discussed between all authors but was not registered or published prior to execution. In line with recommendations [40], the following databases were searched by the lead author in September 2022: Web of Science Core Collection, Scopus, OVID (Embase, MEDLINE, PsycINFO, Global Health, and Health and Psychosocial instruments), and EBSCOhost (Academic Search Complete and Cumulative Index to Nursing and Allied Health Literature Complete). Individual search strategies were created for each database with search terms mapping onto five main areas of interest (see Table 1 for an example of search terms mapped onto the areas of interest).

To create a comprehensive overview of publications relating to each included DHI, we performed both forward (publications citing the article) and backward (publications cited by the article) searches of publications that described a DHI that met or could meet our inclusion criteria. In some cases where the originally retrieved publication was ambiguous in terms of eligibility (eg, not enough detail describing the DHI), forward and backward searches enabled us to reach a conclusive decision.

**Table 1:** Search terms used for the Web of Science database mapped onto areas of interest.

Area of interest	Search terms^a^
1. Digital technology	online OR digital OR internet OR web* OR mHealth OR eHealth OR mobile*
2. Focus on health, health-related lifestyle, or long term noncommunicable diseases	health OR “long term condition*” OR “long term ill*” OR “chronic disease*” OR “chronic* ill*” OR “chronic condition*” OR “non communicable” OR noncommunicable OR NCD* OR comorbid* OR lifestyle
3. Focus on behavior change or self-management	self-manag* OR self-monitor* OR behavio* NEAR/1 change OR lifestyle NEAR/1 change OR modif* NEAR/1 behavio* OR modif* NEAR/1 lifestyle OR health NEAR/1 *manag* OR condition NEAR/1 *manag* OR disease NEAR/1 manag* OR behavio* NEAR/1 manag* OR improv* NEAR/1 health OR improv* NEAR/1 lifestyle OR “behavio* intervention” OR “lifestyle intervention”
4. Resource provides an assessment of current health, health-related behaviors, or lifestyle	health* NEAR/1 assess* OR lifestyle NEAR/1 assess* OR risk* NEAR/1 assess OR health* NEAR/1 evaluat* OR lifestyle NEAR/1 evaluat* OR risk* NEAR/1 evaluat*
5. It provides personalized support in the selection of health-related priority goals	prioriti* OR select* OR choose OR “decision support” OR “decision tool*” OR “decision aid*” OR “competing goal*” OR “competing behavio*” OR “focal goal*” OR “focal behavio*” OR “traffic light” OR recommend* OR advi* OR decid*

^a^The search terms were combined as follows: 1 AND 2 AND 3 AND 4 AND 5. The search field was set to Topic for each row of search terms.

### Eligibility Criteria

To be included, publications had to be in English and be peer-reviewed. Furthermore, publications had to adhere to our eligibility criteria aligned with an adapted version of the Population, Intervention, Context, Outcome approach [41,42].

#### Population

To be included, a key function of the DHI had to be support of users with health self-management. As a result, any DHIs primarily providing support to people other than the person being assessed (eg, a resource providing support to health care providers or carers by assessing patients) were excluded.

#### Intervention

The DHI had to provide its users with prioritization support. This had to include both assessment and prioritization components, with both components covering primary, secondary, or tertiary preventive health behaviors or goals (eg, physical activity, sleep, alcohol use, or stress management) and/or health behaviors associated with managing long-term health conditions (eg, medication adherence, treatment adherence, or condition monitoring tasks). Specific requirements for each component are listed below.

Assessment across multiple domains: The DHI had to assess health-related behaviors across at least two health domains (eg, physical activity and dietary health, or alcohol use and sleep). We use “domain” to refer to a group of health-related behavior patterns relevant to a particular health outcome. For example, the domain “sleep” may include both responses to sleep disruption as well as sleep-hygiene behaviors. Similarly, “physical activity” may include both exercise and sedentary behaviors. In determining DHI inclusion eligibility, we focused on domains, rather than specific behavior patterns, because behavioral goals within the same domain are typically “complementary” rather than competing [43]. Consequently, there is less of a need to prioritize among them. For example, focusing both on increasing exercise and decreasing sedentary behaviors does not create goal conflict. In summary, we excluded DHIs that only assessed behavior patterns in one domain (eg, only physical activity–related behaviors). In addition, we excluded DHIs that provided support across multiple domains but did not assess users in more than one domain.

Prioritization support: To be included, DHIs had to assist the user in selecting and prioritizing health domains or associated behaviors. To enable inclusion of as many DHIs as possible, we allowed this to be very basic. For example, providing assessment-based feedback regarding which assessed health domains or associated behaviors needed attention (eg, using a traffic light system). More elaborate prioritization support could include helping the user select priority goals based on assessment of health and health behaviors, motivational readiness, personal barriers, and preferences. This means that DHIs were excluded if they did not, at the very least, use health assessment data to provide the user with personalized advice or feedback on which health domains or associated behaviors posed a user-relevant health risk. Further, the need to directly link prioritization support to assessment meant that DHIs that provided prioritization advice on health domains or behaviors without first assessing them (eg, advising users to increase exercise and improve diet without having assessed users’ current exercise and dietary behaviors) were also excluded.

#### Context

The assessment and prioritization components of the DHIs had to be digital in nature. When a DHI employed a combination of automated digital support and in-person or digital person-based support (eg, telehealth or an online coach), only those DHIs where the assessment and prioritization components could each be completed in a fully automated manner, albeit with missing data (eg, biometric data that can only be collected by a clinical professional, such as blood glucose or cholesterol assessments), were included. Thus, DHIs in which *either* assessment *or* prioritization support could not be completed without involvement of another person were excluded, even when this interpersonal support was provided through an online or telehealth platform. However, DHI components following the prioritization stage could be digital, in person, or a combination of both.

#### Outcome

Our objective was to review *developed* DHIs that met our inclusion criteria, regardless of study design or article type. We included any publication that (1) reported on the development of a DHI if it had resulted in a functioning DHI that met our inclusion criteria, (2) reported on a current or prospective evaluation or implementation of an existing DHI that met our criteria (eg, feasibility and acceptability studies, randomized controlled trials (RCTs), economic evaluations, protocol papers for existing resources, and implementation papers), or (3) reported on a DHI meeting our criteria, even if the DHI was not the primary focus of the study.

We excluded review papers and conceptual papers as well as publications that failed to provide evidence that the DHI was operational. For example, projections of future developments.

### Screening and Selection

**Figure 1:**
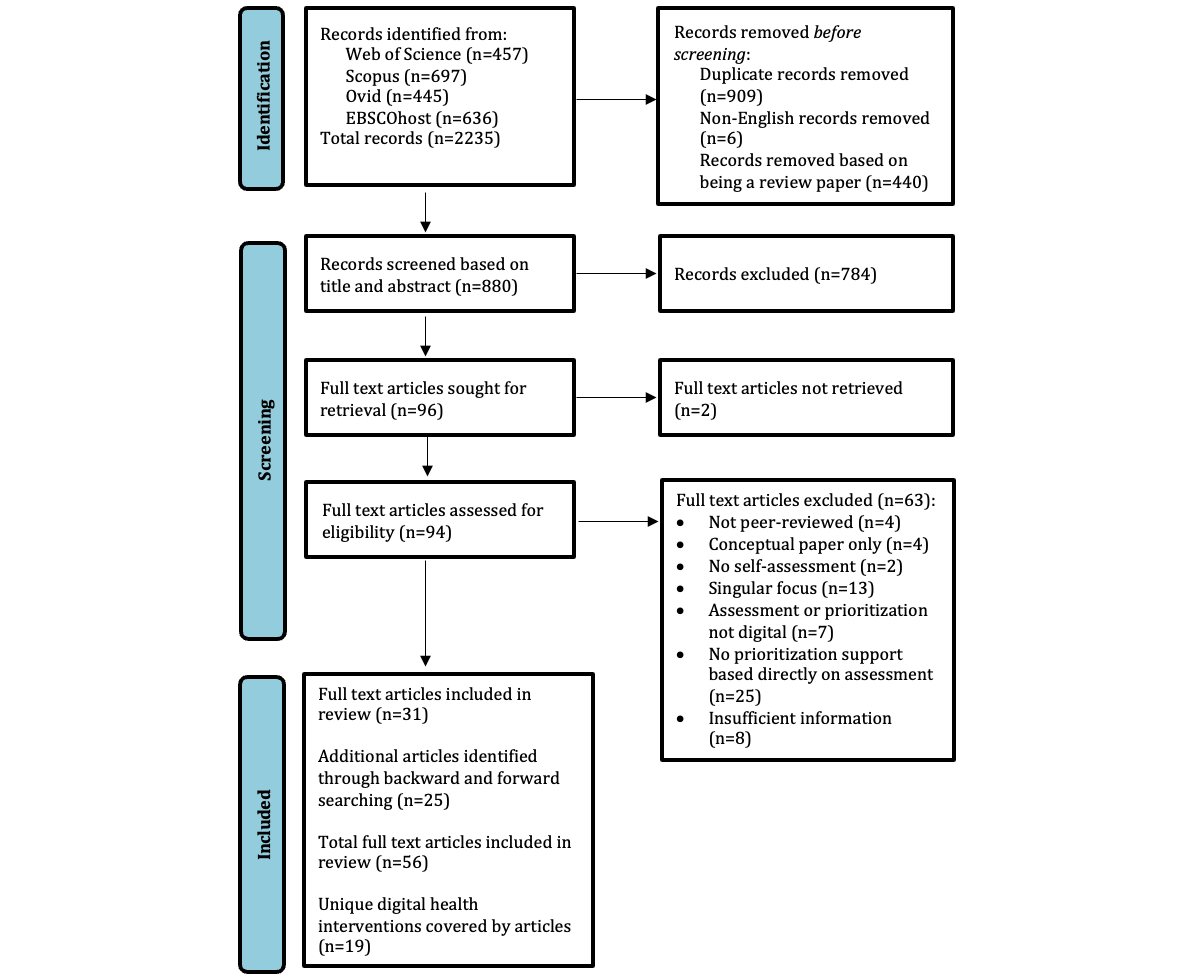
PRISMA (Preferred Reporting Items for Systematic Reviews and Meta-Analyses) flowchart.

Results from each database were exported to a reference manager (EndNote v20.6, Clarivate Analytics). Prior to screening for eligibility, duplicates, publications in a language other than English, and publications that were identified as scoping reviews, systematic reviews, or meta-analyses were removed. Each stage of the screening process is presented in Figure 1. Titles, abstracts, and full texts were all assessed by IM. During the title and abstract stage, 40% of publications were independently screened by CA, while during the full-text article screening stage, 10% of publications were independently assessed by CA. All disagreements were resolved through discussion.

### Data Extraction and Charting

For each included publication, data was extracted by IM into a spreadsheet. First, the following information was extracted from each publication to identify and map publications relating to the same DHI: first author surname, year of publication, article title, journal, and name of the DHI (where available). Then, at the article level, the following additional data were extracted and charted in Table 2: the focus of the publication (ie, development, protocol, or evaluation) and, for evaluation reports, the type of evaluation (ie, process, outcome (including RCT: yes or no), or economic evaluation). Next, at the DHI level, the following data were extracted: (a) country of development (based on the country of the first author or, when unavailable, primary contact); (b) technology platform used; (c) details on how data privacy was handled; (d) target population; (e) development, including theoretical underpinning; (f) assessed behavioral health domains as well as other assessed health-related factors, including factors that might inform the level of health risk across different areas of health (eg, psychological health factors, social health factors, biometric data, and family history); (g) description of prioritization support; (h) support beyond prioritization; and (i) use of prompts to encourage the user to revisit the DHI or continue goal pursuit. Component g (prioritization support) was charted in relation to two criteria. The first was feedback provided on assessment results, which we regarded as the most basic level of prioritization support, where DHIs provided personalized information about which domains or behaviors may be more or less in need of change. Second, we identified DHIs that explicitly prompted or assisted the user in selecting a particular health domain or behavior pattern as a priority for change. Finally, for outcome evaluations that used, at minimum, a pre-post (AB) design, the key findings of that evaluation were charted. Impact measures for effectiveness were classified as significant improvements in health behaviors, physiological health outcomes, psychological health outcomes, social or environmental health outcomes, or antecedents of health outcomes (eg, knowledge and skills). Note that we do not report outcome data based on postintervention assessment when no preintervention or cross-condition comparison was available.

**Table 2:** Overview of included publications (n=56) mapped onto each digital health intervention (n=19).

Name of digital health intervention and corresponding publications		Focus of publication (multiple categories possible)^a^						
		P	D	PE	OE	RCT	EE	SA
**A. Adolescent Diabetes Needs Assessment Tool (ADNAT)**								
	[44]		x					
	[45]^b^			x	x			
**B1. Alive!**								
	[46]				x	x		
	[47]^b^				x	x		
**B2. Alive PD** ^c^								
	[48]^b^	x						
	[49]^b^			x	x	x		
	[50]^b^				x	x		
**C. Carolina Health Assessment Research Tool (CHART)**								
	[51]			x				
**D. Connection to Health**								
	[52]		x	x	x	x		
**E. eVida Toolkit**								
	[53]		x					
**F. Gabby**								
	[54]^b^		x	x				
	[55]			x	x	x		
**G. Health Map**								
	[56]			x				
**H. Healthy Lifestyle Support Program**								
	[57]^b^			x				
	[58]			x	x	x		
	[59]			x	x	x		
**I. Kanker Nazorg Wijzer (** ***Cancer Aftercare Guide*** **)**								
	[60]^b^	x	x					
	[61]			x				
	[62]				x	X		
	[63]^b^				x	X		
**J. Mijn Gezond Gedrag (** ***My Healthy Behavior*** **)**								
	[64]^b^	x						
	[65]			x				
	[66]			x	x	x		
	[67]						x	
	[68]			x				
	[69]			x				
**K. My Health Matters! (MHM) with @live Health Risk Assessment**								
	[70]			x	x		x	
**L. OncoKompas**								
	[71]		x	x				
	[72]^b^	x						
	[73]			x				
	[74]^b^			x				
	[75]^b^	x						
	[76]^b^	x						
	[77]^b^			x	x	x		
	[78]^b^				x			x
	[79]^b^						x	
	[80]^b^			x				
	[81]^b^			x				
**M. Perfect Fit**								
	[82]^b^	x						
	[83]				x	x		
**N. Prevention Compass**								
	[84]			x	x^d^			x
	[85]				x			
	[86]^b^			x				
	[87]	x						
	[88]			x				
	[89]			x				
**O. Prevent Connect**								
	[90]			x				
**P. PRO-FIT**								
	[91]^b^	x						
	[92]			x	x	x		
	[93]^b^			x	x	x		
	[94]^b^			x				
**Q. PULSE (Canada)**								
	[95]		x					
	[96]		x	x				
**R. SPRITE**								
	[97]	x						
	[98]^b^				x	x		
**S. Unnamed HRA** ^e^								
	[99]			x	x^d^			

^a^D: development; P: protocol; PE: process evaluation (including feasibility and acceptability); OE: outcome evaluation; RCT: randomized controlled trial (identified subtype of OE); EE: economic evaluation; and SA: secondary analysis.

^b^Found through a forward or backward search.

^c^Tailored version of the Alive! intervention targeting adults with prediabetes.

^d^These outcome evaluations only included a postintervention evaluation and therefore were excluded from the outcome evaluation results in Table 5.

^e^HRA: health risk assessment.

## Results

### Study Selection

Database searches were executed on September 22, 2022. Across databases, the searches identified 2235 records. After removing duplicates, non-English records, and review papers (identified through the Endnote search function and then manually verified as being scoping reviews, systematic reviews, or meta-analyses), 880 records remained. These records were screened by IM based on title and abstract. Then CA screened 360/880 records, yielding a satisfactory inter-rater reliability (κ) of .84. Had less agreement been found, we would have conducted further, independent intercoder reliability checks. For the 24 publications that resulted in disagreement between authors based on title and abstract assessment, agreement was reached through detailed discussion.

Of the 96 publications retained for full-text screening, 94 were successfully retrieved. IM screened each article and conducted a forward and backward search on any articles describing a DHI that met or could meet our inclusion criteria to identify additional publications reporting the same DHI. Next, CA screened a randomly selected 10% (n=9) of retrieved articles. The reviewing authors independently agreed on all but one article and then agreed on the last one through discussion. A further seven full-text publications that were initially to be screened only by the lead author contained interventions that were judged to be ambiguous in terms of their eligibility. Hence, these publications, and any publications associated with the same DHI found through forward and backward searches, were also coevaluated by CA, with agreement reached on all.

Overall, screening resulted in 31 of the retrieved full text articles being included and 63 being excluded. The primary reasons for exclusion are reported in Figure 1. An additional 25 articles relating to the DHIs covered by the 31 articles included from the search results were found through forward and backward searching, resulting in a total of 56 articles being included. These 56 articles reported 19 unique DHIs, with one DHI containing two versions, one for the general population, and one for a specific subpopulation. Table 2 provides an overview of how the 56 included publications were related to each of the 19 DHIs based on their reporting focus, categorized in terms of whether they reported DHI development, a protocol for evaluation, a process evaluation (including feasibility and acceptability), an outcome evaluation, an economic evaluation, or a secondary analysis [100]. Each DHI was given a unique letter-based identifier (A-S).

### Intervention Characteristics

Characteristics of the 19 DHIs are presented in Table 3. Eight were developed in the Netherlands (H, I, J, L, M, N, P, and S), five in the United States (B, C, D, F, and R), two in Canada (K and Q), and one in each of Australia (G), France (O), Portugal (E), and the United Kingdom (A).

The most common DHI delivery-mode was via a website (n=16). Three (A, H, and O) were delivered through a mobile app. One DHI combined website delivery with email (B) and another mentioned use of a conversational agent (F). A small majority (11/19) of DHIs were said to have taken account of data privacy and security issues (A, B, E, I, J, K, L, N, P, S, and R). Seven DHIs (A, I, J, L, N, P, and S) used a username with password combination for protected access, and one (E) stated that all data were recorded in an anonymous manner. One mentioned use of a firewall (N), and four mentioned being compliant with government or industry standards or regulations (B, E, K, and R).

More than half (n=10) of the 19 DHIs were developed to target the general population (B1, C, D, E, H, J, N, O, Q, and S), with three of these being delivered within a health care setting (D, Q, and S). The remainder targeted populations at risk of or diagnosed with cardiovascular disease (n=4; K, M, P, and R), diabetes (n=2; A and B2), cancer (n=2; I and L), or HIV (n=1; G). One DHI targeted preconception, African American women (F).

**Table 3:** Overview of included interventions (n=19).

Name of DHI^a^	Country	Platform	Data privacy or security mentioned	Target population	Theoretical basis	Assessed behavioral domains and health factors
A. Adolescent Diabetes Needs Assessment Tool (ADNAT)	UK	Mobile app	Yes	Type 1 diabetes	Complexity science, including transtheoretical change cycle	Physical activity; diet; monitoring of blood glucose levels; medication taking; living with diabetes.
B1. Alive! B2. Alive PD^b^	USA	Email, website, and mobile phone (latter for B2 only)	Yes	B1. General population B2. Adults with prediabetes	Health belief model, the theory of reasoned action, social cognitive and social learning theories, goal-setting theory, social marketing, and the transtheoretical model	Physical activity; diet (ie, fruits and vegetables, saturated and trans fats, and added sugars).
C. Carolina Health Assessment Research Tool (CHART)	USA	Website	—^c^	General population	—	Physical activity; diet; alcohol use; tobacco use; weight.
D. Connection to Health	USA	Website	—	General population in primary care setting	—	Physical activity; diet; smoking; alcohol; depressive symptoms.
E. eVida Toolkit	Portugal	Website	Yes	General population	—	Physical activity; diet; smoking; drinking; use of illicit substances; sleep (ie, habits and quality); well-being, social cohesion, and functional independence; mental health; health and disease; anthropometric assessment and cardio-metabolic parameters.
F. Gabby	USA	Website with conversational agent	—	African American women in preconception stage	Provider-patient communication theory	Physical activity; diet (including pregnancy-related nutrition); alcohol; tobacco; illegal substances; emotional and mental health; genetic health; reproductive health; health care risks (eg, insurance); health conditions and medications; immunizations; infectious disease risk; partner health; relationship risk (ie, abuse); environmental risk (eg, toxoplasmosis, well water).
G. Health Map	Australia	Website	—	HIV-positive people	—	Physical activity; alcohol; smoking; psychological health; adherence to antiretroviral therapy; monitoring CD4 T-cell count and viral load (HIV) testing; hepatitis A (HAV) and B (HBV) vaccination status; HBV and hepatitis C (HCV) investigation status; fasting blood cholesterol, fasting blood sugar, blood pressure, and BMI; cervical cytology in women; sexually transmissible infection (STI) screening in men who have sex with men.
H. Healthy Lifestyle Support Program	Netherlands	Mobile app	—	General population	HAPA^d^, I-Change^e^ model	Physical activity; smoking; alcohol; food; energy and recuperation.
I. Kanker Nazorg Wijzer (*Cancer Aftercare Guide*)	Netherlands	Website	Yes	Cancer survivors	Theory of planned behavior, self-regulation theory, and the I-Change model.	Physical activity; diet; smoking; fatigue; return to work; mood; relationships; residual symptoms.
J. Mijn gezond gedrag (*My Healthy Behavior*)	Netherlands	Website	Yes	General population	I-Change model	Physical activity; smoking; alcohol; diet (fruit and vegetable consumption).
K. My Health Matters! (MHM) with @live Health Risk Assessment	Canada	Website	Yes	Working population at risk of cardiovascular disease	—	Physical activity; smoking; alcohol; diet; stress; weight; cholesterol, blood pressure; diabetes status; medication (yes or no) and clinical values if known; personal health history; family health history.
L. OncoKompas	Netherlands	Website	Yes	Cancer survivors or incurable cancer	—	Physical activity; alcohol; smoking; diet; weight; mental health; physical health, such as pain, sexual health, and sleep; social health; specific head and neck cancer–related assessments (see Table 1)
M. Perfect Fit	Netherlands	Website	—	Working population at risk of cardiovascular disease	Transtheoretical model	Physical activity; diet; smoking; alcohol; work (stress); family history; medical history; blood, anthropometric, and biometric measures.
N. Prevention Compass	Netherlands	Website	Yes	General population	Transtheoretical model, protection motivation theory, and social cognitive theory	Physical activity; smoking; alcohol; diet; psychological health (stress, burnout, depression); sociodemographic variables; family and personal medical history.
O. Prevent Connect	France	Mobile app	—	General population	—	Unhealthy eating; sedentary lifestyle; alcohol; tobacco.
P. PRO-FIT	Netherlands	Website	Yes	Adults with familial hypercholesterolemia	I-Change model (2.0)	Physical activity; diet (fruit, vegetables, and saturated fat intake); smoking; compliance to statin therapy.
Q. PULSE	Canada	Website	—	General population in primary care setting	Transtheoretical model	Physical activity; smoking; diet; alcohol; stress; depression; age; blood pressure; glycemic control values. Additional measure: behavior change readiness for elements not in accordance with accepted targets.
R. SPRITE	USA	Website	Yes	Adults who have been diagnosed with acute MI^f^ and hypertension	Health decision model, which is based on the health belief model, and transtheoretical model	Physical activity; diet; smoking; alcohol; stress reduction; memory; literacy; social environment; patient-provider relationship; missed appointments; medication management; side effects; knowledge and risk perception; cardiovascular risk.
S. Unnamed HRA^g^	Netherlands	Website	Yes	General population in care setting	Transtheoretical model, protection motivation theory, and social cognitive theory	Physical activity; alcohol; smoking; cardiovascular and psychological risk.

^a^DHI: digital health intervention.

^b^ This was a tailored version of the original Alive! Intervention.

^c^No information on this element was found across publications.

^d^HAPA: health action process approach.

^e^I-Change: integrated change model.

^f^MI: myocardial infarction.

^g^HRA: health risk assessment.

### Theoretical Underpinnings

Of the 19 DHIs, 12 had clear theoretical underpinnings (Table 3). Many claimed to have applied psychological theories such as the transtheoretical model (n=7; A, B, M, N, Q, R, and S), social cognitive theory (n=3; B, N, and S), health belief model (n=2; B and R), protection motivation theory (n=2; N and S), health action process approach (n=1; H), theory of planned behavior (n=1; I) or theory of reasoned action (n=1; B), goal setting theory (n=1; B), self-regulation theory (n=1; I), and the overarching integrated change model (n=4; H, I, J, and P). One DHI report referred to social marketing theory (B), one referred to provider-patient communication theory (F), and another referred to complexity science as a guide for intervention development (A). For seven (of 19) DHIs, there was no reference to theoretical underpinnings.

### Design Processes

We were able to find details on DHI development or design processes for just seven of the 19 DHIs (A [44], D [52], E [53], F [54], I [60], L [71], and Q [95,96]). Five out of these seven mentioned involvement of members of the user population at one or more stages of the development (A, D, F, I, and L), whereas two did not (E and Q). Among the five involving users, two (I and L) reported conducting an early stage needs assessment before commencing development of the DHI. To guide the development process, one DHI (I) was developed using intervention mapping [101] and another (A) was developed using recommendations for the design and validation of questionnaires [102,103].

### Assessed Health Domains

Health domains as well as other health factors assessed by the DHIs (components a-f from the Data Extraction and Charting section) are reported in Table 3. All (n=19) assessed physical activity. Most (n=17) assessed diet (all except G and S) and smoking (all except A and B), closely followed by alcohol use (n=15; all except A, B, I, and P). However, only two DHIs mentioned assessment of illegal substance use (E and F). Four assessed sleep (E and L) or associated constructs (fatigue: I; energy levels: H). One assessed pain (L). The four DHIs involving populations receiving medical treatment (A, G, P, and R) all assessed medication adherence, with one DHI assessing several other factors related to treatment (ie, patient-provider relationship, missed appointments, and side effects of medication; R).

Other health-related factors were also assessed; 13 DHIs assessed at least one factor related to psychological health (eg, stress, depression, mood, or well-being; A, D, E, F, G, I, K, L, M, N, Q, R, and S) and five assessed at least one social-environmental factor, such as social isolation or risk of domestic violence (E, F, I, L, and R). To enable further personalization of feedback based on risk profiles, six DHIs included an anthropometric assessment (eg, weight or BMI; C, E, G, K, L, and M) and seven included cardio-metabolic parameters (eg, cholesterol, blood pressure, or glycemic levels; E, G, K, M, Q, R, and S). Five assessed personal medical history (E, F, K, M, and N) and three assessed family medical history (K, M, and N).

### Prioritization Support

The extent of prioritization support through prioritization feedback and subsequent prioritization assistance as well as any postprioritization support offered by each DHI (components g-i from the Data Extraction and Charting section) are reported in Table 4. Prioritization feedback to users took several different forms. Nine (of 19) DHIs included a traffic light–based feedback tool, which used color coding (eg, green, orange, and red) to indicate the extent to which each of the assessed health domains or associated behaviors presented a health risk or was in need of change (A, E, I, J, L, M, N, O, and S). For most of these (7/9; all except A and I), traffic light information was combined with written feedback. The non–traffic light–based DHIs (10/19) all provided written feedback. Written feedback generally included information on how each domain or associated behavior differed from national guidelines or was related to the user’s health risk levels. For some DHIs (n=7), feedback included suggestions on how to reduce health risk or create change in high-risk domains (C, F, N, O, P, Q, and S). One DHI included specific information on clusters of interrelated factors that showed elevated risk levels (L).

**Table 4:** Overview of prioritization and behavior change support elements of included interventions (n=19).

DHI^a^	Prioritization support	Additional support past prioritization	Prompts to revisit DHI or continue goal pursuit
A.	Feedback: Traffic light feedback on assessment scores, with red indicating high need, orange indicating intermediate need, and green indicating low need.	—^b^	No
B1.	Feedback: Separate reports on assessment domains. Prioritization: Participants choose an initial module to work on for the subsequent 3 months.	Participants receive weekly messages offering tailored small-step goals to choose for the following week, tailored tips for achieving those goals, health information, and numerous opportunities for interaction and engagement.	Weekly emails.
B2.	Feedback: Separate reports on assessment domains. Prioritization: Participants are prompted to choose at least one physical activity and one dietary goal.	As B1, with added detail: the intervention offers social support through a team system, which allows participants to send messages to each other with either a predrafted motivational message or a personalized message of support. Other features include a daily tracker to log weight, activity, and dietary intake, with automatic graphing over time; an automated coaching tool to help participants overcome common barriers; weekly infographics that reinforce core messages of the program; downloadable worksheets; and links to external resources.	Weekly emails.
C.	Feedback: Health summary with tips and suggestions for behavior change.	—	No
D.	Feedback: Written feedback on assessment results with recommendations regarding what health behaviors to modify.	Basic site included educational materials on four health behaviors. Enhanced site also included a section about action plans, prompting users to develop an individualized plan; a discussion forum section, where patients could post issues and discuss them with other patients working on similar behavioral changes; and an “Ask the Expert” section, where patients could pose questions for the clinical team.	Periodically
E.	Feedback: Traffic light feedback on assessment components paired with written recommendations. Low risk: green; moderate risk: yellow; and relevant risk: orange. Further assessment: Individuals showing moderate or relevant risk in one or more dimensions are invited to participate in a more detailed assessment.	—	No
F.	Feedback: Risks are identified in “My Survey Results.” User can select a risk to learn more about. Gabby then provides informational script on risk, followed by questions capturing the stage of change for risk. This results in stage-appropriate guidance and tips for change. Prioritization: The user either adds the risk to the My Health To Do List or selects a different risk to learn about.	Adding a risk to the My Health To Do List results in receiving longitudinal behavior change scripts for that risk.	No
G.	Feedback: Individualized reports highlight health issues of concern and advise users to discuss these with their treating clinician and health care team at subsequent consultations.	Links to and information on a series of relevant websites.	No
H.	Feedback: Automated feedback based on assessment.	Personal action plan drawn up at the start workshop. Use of weekly buddy contacts. Access to mHealth Health Quiz app. Supporting health education materials via email. A 2-hour repeat workshop after 1 month for answering questions and for (peer) education. Twelve times a weekly health tip email to help maintain awareness and motivation.	Weekly
I.	Feedback: Traffic light feedback on assessment scores, with green indicating no need to complete module, orange indicating some potential for improvement by completing module, and red indicating the user is advised to complete this module. Modules are inter-related in that users may be advised to visit several modules simultaneously (eg, fatigue and physical activity) if both need improvement.	The module content consists of two sessions (session 1: problem identification, goal setting, and action planning; session 2: evaluation) and is customized to personal characteristics (gender, age, marital status, children, educational level, BMI), cancer-related issues (type of cancer, type and number of comorbidities), motivational behavioral determinants (attitude, self-efficacy, and intention), and current lifestyle behavior.	Per module: 30 days after session 1 to engage in session 2. Monthly news items.
J.	Feedback: Traffic light (red, orange, green) paired with an explanation for their rating. Respondents can click on links to receive additional information about the guidelines and the specific health behavior. Prioritization (in sequential condition only): Users choose one behavior with orange or red light based on personal motivation.	Based on additional assessment measures, personal advice is provided on selected behaviors. This includes (1) advice targeting attitude change, (2) advice on social influence and how to navigate it, (3) advice on forming an action plan, and (4) advice to increase self-efficacy and appropriate coping responses.	Respondents were encouraged to revisit website, but no mention of specific prompts.
K.	Feedback: All risk factors are explained in detail and are organized into one of five categories: (1) lifestyle habits (smoking, diet, alcohol, and self-medication); (2) coping factors (stress, exercise, and relationships); (3) environment (toxic materials and sun); (4) health indicators (blood pressure, cholesterol, blood sugar, weight, and age); and (5) family factors. Users also receive an overall health score. The lower the score, the lower the individual’s risk of developing chronic disease; the higher the score, the higher the risk.	Users receive a checklist and action plans for implementing changes to lower their risk factors. Users can also attend one-on-one onsite clinical sessions with registered nurses and partake in onsite educational initiatives to educate them of the risk factors associated with metabolic diseases. Initiatives include lunch and learn sessions, awareness bulletins, monthly emails, newsletters and videos, and activity challenges.	None for the digital component.
L.	Feedback: A compass metaphor is used to summarize overall well-being. Traffic light feedback is provided using green (no elevated well-being risks), orange (elevated well-being risks), and red (seriously elevated well-being risks). In addition, users receive elaborated personalized information on the outcomes, including information on clusters of interrelated factors when these are elevated.	Users are provided self-care advice (tips and tools) tailored to the individual user. In the DHI component, users are provided with personalized supportive care options, based on their participant reported outcome scores and expressed preferences (eg, preference for individual therapy versus group therapy).	No
M.	Feedback: Traffic light profiles (green: low risk; orange: intermediate risk; or red: high risk). Prioritization: A suggestion to make a choice out of several health promotion activities based on the participant’s risk profile, preferences, and motivational aspects.	In the Motivational Interviewing (MI) condition, personalized MI sessions with an occupational physician were added, together with additional tailoring based on motivational elements in the web-based HRA and an additional motivational paragraph in the newsletters.	No
N.	Feedback: Traffic light profiles (green: normal risk; orange: moderately elevated risk; red: seriously elevated risk) divided into four domains (lifestyle, psychological, social, and physical) with subdomains. Threats associated with elevated risk (orange and red) and potential gains of taking preventive action are explained. The feedback concludes with an overview of actions the participant can take to address elevated risk categories.	Personalized suggestions are made to support actions based on the stage of motivation, the preferences for professional guidance (yes or no), actions in groups or alone, actions outside or close to home, and actions via internet, telephone or face-to-face. In case of high risk (red category), the feedback includes referral for further medical evaluation and treatment if necessary.	No
O.	Feedback: Each assessed behavior is presented with an arrow on a color scale ranging from green (“very good behavior”) to red (“very bad behavior”). Underneath the arrows, the app provides recommended actions for each behavior in need of change.	Following the feedback screen, users go to a screen that displays DHIs that would be helpful in encouraging change in the behaviors that require it.	No
P.	Feedback: Based on assessment the user receives (1) feedback on how each behavior contributes to their overall CVD^c^ risk, (2) information on the changeability of the behavior, and (3) cues about how the risk behavior may be changed.	Users are provided access to face-to-face counselling complemented with telephone booster sessions (PRO-FIT coach).	None for the digital component.
Q.	Feedback: Each risk factor has its own feedback section, including an introductory brief, the patient’s current results, evidence-based target values, lifestyle modifications, and risk man­agement education. Feedback is personalized based on current behavior and motivational readiness for change.	—	No
R.	Feedback: Tailored feedback to reinforce evidence-based behavior for disease and lifestyle management.	—	Monthly assessments and information modules (digital arm).
S.	Feedback: Tailored health action plans, where each plan contains (1) the outcome of the individual risk assessment using a traffic light system (green: normal risk; orange: moderately elevated risk; red: seriously elevated risk), (2) an explanation of the associated health risks and potential gains of preventive action, (3) individual opportunities for lifestyle change, based on motivation, self-efficacy, and preferences.	The participant receives referral suggestions and information for either local providers with individual or group activities or online offers. Follow-up actions include lifestyle advice and starting with (preventive) drug treatment (eg, lipid and blood pressure lowering drugs).	No

^a^DHI: digital health intervention.

^b^No information on this element was found across publications.

^c^CVD: cardiovascular disease.

Although each of the 19 DHIs included some form of algorithm-based feedback on which domains or associated behaviors required change, only three provided further prioritization support by prompting the user to make a choice as to which behaviors they wanted to start working on (B, F, and J). One other (M) suggested the user should make a choice, but it is unclear whether this choice was recorded by the DHI. All feedback was based on user assessment results, but only five DHIs further personalized feedback based on psychological factors related to behavior change, such as stage of change, attitudes towards change, self-efficacy, and coping plans (F, J, M, P, and Q).

### Postprioritization Support

Most DHIs (14/19) provided additional support beyond the feedback stage. However, the form of this additional support varied widely. Some (n=6) offered integrated in-person support (H, M, and P) or referrals or recommendations for in-person support (L, N, and S). Several (9; B, D, F, H, I, J, K, L, and M) included integrated change support of a digital nature (eg, online modules and progress trackers), and others (n=4) provided links to useful external digital resources (eg, online health education) or other DHIs (G, N, O, and S). Five DHIs did not provide any support beyond feedback provided on the assessment (A, C, E, Q, and R).

### Prompts to Revisit DHIs

The majority of DHIs (n=12) did not include prompts to enable users revisit the DHI for support after an initial interaction (A, C, E, G, K, L, M, N, O, P, Q, and S). One DHI may have included revisit prompts, but the nature of these was unclear (J). Out of the six DHIs that explicitly mentioned prompting users to revisit (B, D, F, H, I, and R), only one prompted users to revisit the DHI with a view to assessing progress toward or revision of change goals (D).

### Outcome Evaluations

Outcome evaluations that used, at minimum, a pre-post (AB) design are reported in Table 5. Most DHIs (14/19) reported at least one outcome evaluation (22 evaluations in total), with ten reporting at least one RCT (15 RCTs in total; see Table 2). Amongst the five DHIs not reporting any outcome evaluations (C, E, G, O, and Q), one mentioned plans to conduct an RCT [90], but no results had been published at the time of writing. Out of 14 DHIs reporting at least one outcome evaluation, one (S) was only evaluated cross-sectionally after the intervention had been completed, without a comparison condition. The evaluation of this DHI is excluded from the results presented in Table 5.

**Table 5:** Characteristics and results from outcome evaluations.

DHI^a^	Study design	Target group, n participants; mean age (years)	Data collection	Outcome categories	Findings
A. [42]	Pre-post comparing ADNAT^b^ completers vs noncompleters	89 young people with type 1 diabetes aged 12-18 years; 14.3 for completers, 14.5 for noncompleters	Baseline-6 months	Physiological	HbA_1c_^c^: greater reduction for completers than noncompleters.
B1. [43]^d^	RCT^e^ with waitlist control	787 nonmedical employees of Kaiser Permanente of Northern California; 44	Baseline-4 months	Psychological health, antecedents	The following outcomes showed significantly greater improvement for the intervention group than the control group: quality of life, mental score, self-assessed health status, presenteeism at work, self-efficacy to change diet, self-efficacy to change physical activity, stage of change for physical activity and diet.
B1. [100]^d^	As above	As above	As above	Behavioral	The following outcomes showed significantly greater improvement for the intervention group than the control group: physical activity, diet (greater decline in intake of both saturated and trans fats and greater increase in consumption of fruits and vegetables in the intervention group). There was a marginal effect for the consumption of added sugars.
B2. [45]^f^	RCT with waitlist control	339 patients in an ambulatory care health care delivery system whose recent fasting glucose or HbA1c were within the prediabetes range 55	Baseline-6 months	Physiological	The following showed greater reduction in the intervention group than the control group: fasting glucose, HbA1c, weight, BMI, waist circumference, and Framingham 8-year diabetes risk. In addition, the intervention group showed a greater increase in TG^g^/HDL^h^ ratio than the control group.
B2. [46]^f^	As above	As above	As above	Behavioral, psychological health, antecedents	The following outcomes showed significantly greater improvement for the intervention group than the control group: diet (greater intake of fruits and vegetables and reduction of refined carbohydrates), physical activity, antecedents (self-rated health, self-efficacy to change diet, ability to concentrate, ability to resist illness), fatigue.
D. [48]	RCT with active control	169 patients aged 18-65 years; 43	Baseline-3 months-6 months	Behavioral, psychological health	No differences were found between the intervention and control groups for diet (marginal), physical activity, total number of health risks, depression, or quality of life. For smoking- and alcohol-related outcomes, there were not enough participants to run analyses.
F. [51]	RCT	100 women who self-identified as African American or Black, were 18-34 years of age, and self-reported not being pregnant at enrolment; 25.5	Baseline-6 months	Behavioral, psychological health, social or environmental health	Overall risks resolved: a higher number and percentage of reported risks were resolved in the intervention group than the control group. A higher percentage of triggered risks was resolved in the intervention group than the control group for emotional or mental health, men and health care, and nutrition and activity. No differences between the intervention and control groups were found for environmental risks, genetic health history risks, health care risks, health condition or medicine risks, relationship risks, reproductive health risks, substance use, or immunization or vaccine risks (marginal).
H. [54,55]	RCT with waitlist control	116 employees with cardiovascular disease; N/A^i^	Baseline-6 weeks	Physiological	The following showed greater improvement for the intervention group than the control group: total cholesterol, LDL^j^, and weight. No differences were found between the intervention and control groups for HDL, systolic blood pressure, diastolic blood pressure (marginal), glucose, or HbA1c.
I. [58]^k^	RCT with waitlist control	462 cancer survivors diagnosed with various types of cancer and who had completed primary cancer treatment (surgery, chemo-, or radiation therapy) with curative intent at least 4 weeks and up to 56 weeks prior to initial participation; 55.9	Baseline-6 months-12 months	Behavioral	Baseline to 6 months: Physical activity: no differences between intervention and control groups. Diet: vegetable intake improved more in the intervention group than the control group. Baseline to 12 months: Physical activity: moderate physical activity improved more in the intervention group than the control group. Diet: no differences in vegetable intake between the intervention and control groups.
I. [59]^k^	As above	As above, with mean age now calculated across those completing 6 months of follow-up; 56.3	As above	Psychological health, social or environmental health	The following showed greater improvement in the intervention group than the control group: depression, fatigue, emotional functioning, and social functioning. No differences were found between the intervention and control groups for global health status, physical functioning, role functioning, cognitive functioning, or anxiety.
J. [62]	RCT with simultaneous vs sequential goal engagement vs control	5055 adults aged 18-65 years with Internet access and basic Internet literacy as well as a valid email; 44.2	Baseline-12 months-24 months	Behavioral	No differences at 12 or 24 months: Physical activity and diet. Simultaneous vs control: Overall risk at 12 months: no difference. Overall risk at 24 months: simultaneous group saw greater reduction than the control group. Alcohol and smoking at 12 or 24 months: no difference. Sequential vs control: Overall risk at 12 months: sequential group saw greater reduction than the control group. Alcohol and smoking at 12 months: no difference. Overall risk at 24 months: no difference. Alcohol at 24 months: sequential group saw greater reduction than the control group. Smoking at 24 months: no difference. Simultaneous vs sequential: Overall risk at 12 or 24 months: no difference. Alcohol at 12 or 24 months: no difference. Smoking at 12 and 24 months: sequential group was better than the control group.
K. [66]	Pre-post 1-armed trial	253 employees with at least 1 risk factor for metabolic syndrome; N/A	Baseline-6 months	Physiological	Significant reductions in blood pressure, cholesterol, and total risk factors. No reduction in blood glucose or waist circumference.
L. [73]^l^	RCT with waitlist control	625 cancer survivors diagnosed with head and neck cancer, colorectal cancer, breast cancer, Hodgkin lymphoma, or non-Hodgkin lymphoma; 65	Baseline-1 week-3 months-6 months	Antecedents, social or environmental health, psychological health	No differences were found between the intervention and control groups for knowledge, skills, confidence, mental adjustment, supportive care needs, personal control, patient, and physician interaction. Quality of life showed greater improvement for the intervention group than the control group. For cancer-specific outcomes, see Table 3.
L. [74]^l^	As above	As above	As above	Psychological health, physiological, social or environmental health	The intervention showed the greatest impact on quality of life for cancer survivors with low to moderate self-efficacy and among those with high personal control and those with high health literacy scores.
M. [79]	Randomized trial with two intervention conditions (limited vs extensive)	491 workers in 18 work units from military, police, and a hospital with increased cardiovascular risk; 50.8	Baseline-6 months-12 months	Behavioral, physiological	No differences were found between the intervention and control groups for self-rated health, BMI, body weight, work ability, sickness or absence, physical activity, smoking, or excessive alcohol use.
N. [81]	Pre-post 1-armed trial	368 employees; 45	Baseline-6 months	Physiological	No differences were found pre-post for smoking (marginal), diastolic blood pressure, BMI, or LDL. Significant improvements were found pre-post for systolic blood pressure, waist circumference, total cholesterol, HDL, and TG. Framingham 10-year CVD^m^ risk improved for those with a baseline risk of at least 20%.
P. [88]^n^	RCT with pure control	340 adults with familial hypercholesterolemia; 45.3	Baseline-12 months	Physiological	No differences were found between the intervention and control groups for cholesterol, systolic blood pressure, blood glucose, BMI, or waist circumference.
P. [89]^n^	As above	As above	As above	Behavioral	No differences were found between the intervention and control groups for physical activity, diet, smoking, or compliance to statin therapy.
R. [94]	RCT with active control (education-only) and two intervention conditions (nurse-administered program and web-based interactive tool)	415 adults with myocardial infarction; 61	Baseline-12 months	Physiological	No differences were found between the intervention and control groups for systolic blood pressure, diastolic blood pressure, HbA1c, or cholesterol.

^a^DHI: digital health intervention.

^b^ADNAT: Adolescent Diabetes Needs Assessment Tool.

^c^HbA1c: hemoglobin A1C.

^d^References share the same underlying dataset.

^e^RCT: randomized controlled trial.

^f^References share the same underlying dataset.

^g^TG: triglyceride.

^h^HDL: high-density lipoprotein.

^i^Not available.

^j^LDL: low-density lipoprotein.

^k^References share the same underlying dataset.

^l^References share the same underlying dataset.

^m^CVD: cardiovascular disease.

^n^References share the same underlying dataset.

Out of 22 outcome evaluations, 20 included an outcome evaluation with both pre- and postintervention measurement of outcomes for 13 DHIs. These are listed in Table 5, together with their characteristics and conclusions. We classified outcome measures as behavioral (eg, physical activity and smoking; 10/22), psychological health (eg, quality of life and depression; 8/22), physiological (eg, cholesterol and weight; 9/22), social or environmental health (eg, relationship risk and social functioning; 4/22), or behavior change antecedents (eg, knowledge and self-efficacy; 3/22). Among the 15 RCTs, sample sizes ranged from 100 to 5055, and timing of follow-ups ranged from 1 week [77] to 24 months [66] postbaseline. All but one DHI (D [52]) provided either effect sizes (eg, standardized regression weights and Cohen *d*) or sufficient statistics to calculate them (eg, means and SDs). Effect sizes for significant effects were generally small.

### DHIs With Priority Setting Prompts

Outcome evaluations of the three DHIs that explicitly asked users to select a priority change area (B, F, and J) and the one that suggested doing so (M) all employed RCTs. One of these (B) reported significant effects for the DHI across all outcomes when comparing intervention and control in terms of change from baseline to follow-up (4 months for version B1 and 6 months for version B2). This included both behavioral, psychological, and physiological outcomes [46,47,49,50]. This DHI had a limited focus on behavioral health domains, targeting only physical activity and two types of dietary behavior patterns (ie, consumption of fruits and vegetables and consumption of saturated and trans fats).

Evaluations of the two other DHIs that explicitly prompted the user to record a priority domain (F and J) reported mixed effects. For one (F [55]), the intervention condition resulted in a higher number of health domains or behaviors identified as risks at baseline being resolved at follow-up (ie, no longer being a health risk) compared to the control condition, and some domains and behaviors showed significant differences in resolution between the intervention condition and control over time (eg, nutrition and activity and emotional and mental health), whereas others did not (eg, relationship risks and substance use).

The other DHI (J [66]) that showed mixed effects was evaluated using an RCT that compared a control condition to two DHI versions, referred to as the sequential and simultaneous versions. The sequential version prompted prioritization of a single domain on first use and then again prompted the user to select and prioritize a single domain at 12 months. This version offered support for the prioritized domain during the 12 months following its selection (eg, if physical activity was prioritized at baseline, the user gained access to physical activity–related digital support during the next 12 months until the user was prompted to select another priority at 12 months, at which point they gained access to support for the newly selected domain for the next 12 months). This means participants were able to prioritize and access support for two health domains across the two-year trial period. The simultaneous version did not prompt prioritization. This version offered support across all health domains targeted by the DHI across the full 24 months. Results showed that the sequential version resulted in a greater reduction of overall health risk at 12 months compared to the control condition, but this difference in reduction was no longer significant at 24 months. In contrast, the simultaneous version showed greater overall risk reduction at the 24-month point compared to the control condition, but not at 12 months. Examining differences in terms of the individual health behaviors targeted by the intervention (physical activity, diet, smoking, and alcohol use), the evaluation found that the sequential version resulted in a greater reduction of alcohol use at 24 months as compared to the control condition. Results also showed that the sequential version resulted in a greater reduction of smoking compared to the simultaneous version both at 12 months and 24 months. Overall, this evaluation offered a very mixed set of findings, with the sequential (prioritization) version showing benefits over the simultaneous (nonprioritization) version in certain health domains, but not others. Interestingly, prioritization seemed to be more effective for hard-to-change (or addictive) behavioral patterns. Notably, this was the only evaluation that attempted to assess the potential added value of goal prioritization.

Finally, an RCT of a DHI that suggested users should select a priority, without explicit recording of the priority (M [83]) compared two versions of the DHI: an extensive version, which included both the DHI and coaching, and a limited intervention condition, which only included the DHI, with no coaching. Results showed no significant differences in either behavioral (physical activity, smoking, or excessive alcohol use) or physiological (BMI or body weight) outcomes between the two versions. Both conditions showed a significant improvement in physical activity and reduced excessive alcohol use over time, indicating that the no-coaching version could be more cost effective.

### DHIs Without Priority Setting Prompts

Of the nine evaluated DHIs that provided feedback based on an HRA but did not prompt users to set priorities for change, six (D, H, I, L, P, and R) were evaluated using an RCT. Of these, one reported significant improvement on all physiological outcomes reported from baseline to a 6 week follow-up (H [58,59]), but this DHI was not evaluated on behavioral or psychological outcomes. Another (I) showed mixed results for behavioral outcomes [62], with the DHI resulting in greater dietary improvement than the control at 6 months, but not at 12 months, and greater physical activity at 12 months, but not at 6 months. In addition, this DHI showed significant improvements for the DHI on psychological (depression, fatigue, and emotional functioning) and social (social functioning) outcomes when compared to the control [63]. Finally, a third DHI (L [77]) showed an impact of the DHI on quality of life, but not on any of the other psychological or social outcomes evaluated. However, this DHI did show some additional effects on several cancer-specific outcomes that were targeted and evaluated for relevant subsets of participants.

The remaining three DHIs (D, P, and R) showed no significant outcome effects for behavioral outcomes [52,93] nor for psychological [52] or physiological [92,98] outcomes. Two of these (P and R) compared the DHI condition to a control condition with no HRA feedback, and one compared two DHI conditions (basic versus enhanced) in which HRA-based feedback was provided followed by access to a website with educational materials and tips for behavior change, with the main difference between conditions being that the enhanced condition offered extra support on action planning.

Another three DHIs (A, K, and N) were evaluated through a 1-armed trial. Each of these evaluated only physiological outcomes. One found improvements in the outcome they focused on from baseline to follow-up (HbA1c; A), and one found improvements in the outcome they focused on for high-risk participants only (Framingham cardiovascular disease risk; N). The third (K) found improvements on some (diastolic blood pressure and cholesterol) but not other (BMI and blood glucose) physiological outcomes.

## Discussion

### Principal Findings

This is the first review to systematically identify digital interventions designed to promote self-management of health (DHIs) that contain an assessment of current health or health behavior combined with prioritization support for setting health-related goals. Nineteen DHIs met our inclusion criteria by assessing and providing feedback on health status or health behavior patterns within at least two health domains (eg, physical activity and diet). This is surprisingly few given the size of the digital health global market and the critical importance of goal setting and goal prioritization to successful health behavior change [15,25,26,28,104]. By examining the characteristics of these interventions, their theoretical bases, their design protocols, and their evaluations, this review helps construct a blueprint for the state-of-the-art design of digital interventions intended to guide health behavior change. In particular, we suggest the inclusion of self-regulatory support mechanisms to help users identify and prioritize health-promoting actions as well as engagement mechanisms that promote engagement and re-engagement with prioritized change over time. We argue that these behavior change promotion components need to be carefully embedded in DHI architecture. Finally, we also provide guidelines on how such DHIs might be most effectively evaluated.

Across the 19 DHIs, substantial variation was found in terms of (1) their theoretical bases, that is, the understanding of behavior change and goal pursuit that was used to guide development; (2) the developmental stages and procedures employed (eg, involvement of end-users and theoretical basis); and (3) the components or techniques used to engage users (eg, assessment, prioritization, and postprioritization). Outcome evaluations were disparate in focus and design. Only 8 of 20 evaluations that had, at minimum, a pre-post intervention design included behavioral outcomes, thereby prohibiting conclusions relating to behavior change effectiveness for the majority of DHIs.

The diversity of design, evaluation methodology, and evaluation outcomes prohibit meta-analyses that would generate coherent DHI design recommendations. Nonetheless, the limited number of DHIs including explicit prioritization prompts (n=3; B, F, and J) *all* showed effectiveness in reducing health risk, although this was not always translated into changes across all behaviors or domains nor into the maintenance of change over time. In contrast, evaluations of DHIs *excluding* explicit priority setting prompts generally showed less favorable results, particularly for behavioral outcomes. These indicative findings are consistent with experimental evidence on goal prioritization and behavior change [26].

### DHI Development

The majority of included DHIs (12/19) were developed using theoretical guidance, but there was no consensus on what type of theory was most appropriate. We identified 12 distinct theoretical underpinnings. DHI development is more likely to be effective if founded on evidence-based theories that explain how changes in behavioral antecedents (such as knowledge, motivation, goal setting, and goal prioritization) translate into behavior change [26,101,105,106]. Consequently, consensus on theoretical foundations most likely to generate behavior change would be helpful for future DHI development.

We were unable to confirm whether a stated theoretical basis for development led to greater effectiveness because there was substantial overlap in DHIs lacking theoretical underpinnings and those without outcome evaluations. In addition, many of the DHI reports that highlighted a theoretical bases failed to explain how theory was translated into technique selection and intervention build. An evidence-based theoretical foundation for DHI development with clear intervention design guidance would greatly enhance the research and progressive development of DHIs [107,108].

Five of seven DHIs that reported on development reported user involvement in the design process. This is encouraging because the impact of user involvement in design has been demonstrated [109,110]. Surprisingly only two of these seven DHIs conducted a needs assessment prior to development despite evidence suggesting that such assessments improve intervention effectiveness [101,106,107].

### Behavior Change Targets and Assessment

Included DHIs predominantly addressed a limited set of health-related behavior patterns, in particular, physical activity, diet, smoking, and alcohol use. Only two DHIs assessed and targeted sleep, with two more targeting fatigue or energy levels. This is disappointing given the importance of sleep deprivation to health, such as its impact on the functioning of our immune systems [111,112] and our ability to regulate other health-related behavior patterns [113-115]. Similarly, pain, which was assessed by one DHI only, has been related to both physical and psychological health, and although there is a need for further research into the exact nature of these relationships [116,117], it is clear that pain can both influence and be influenced by other health behaviors and domains, warranting its inclusion in DHIs focused on improving our health and health self-management. Finally, social well-being was targeted by just five DHIs, even though relationships have been shown to affect physical health, mental health, and mortality risk [118-120].

We suggest that there is merit in including broader health and health behavior assessments within DHIs. This does not only ensure that the goal that is prioritized is amongst the most likely to provide benefits to the person but also enables the identification of groups of health behavior patterns that may best be prioritized together. Using interventions to target a greater range of health behaviors and domains has been recommended as the preferred approach to managing noncommunicable diseases given that these conditions are generally complex and influenced by multiple health behavior patterns, so targeting single behaviors is likely to be less effective than targeting a range of behaviors [121,122]. It is noteworthy, that there were only two DHIs (I and L) that identified problem domains that commonly co-occurred and advised users on which domains might benefit from being prioritized or addressed in a conjoined manner (eg, fatigue and physical activity).

### Prioritization Support

For most DHIs, prioritization support was limited to providing feedback that showed users which of the assessed domains needed attention (15/19), with some of these (n=7) including advice on how each of the domains that required attention might be improved through behavior change. In other words, none of these 15 DHIs provided the user with assistance on how to manage multiple behavior change goals by prompting or suggesting the selection of one or more priority goals. Only four DHIs provided such guidance.

### DHIs With Priority Prompts

RCTs of the three DHIs that included prioritization prompts (B, F, and J) suggest that these were more effective than ones without prioritization prompts, but we do not have enough evidence to confirm this trend. Consider an illustrative example. The Mijn gezond gedrag (My Healthy Behavior; J) website included a DHI version that prompts the user to select priorities. This version was more effective than the version that did not prompt prioritization in supporting smoking cessation, both at 12 months and 24 months. This is an impressive finding. Overall, however, the results of this evaluation were mixed, prohibiting clear conclusions. Further RCTs assessing the value of adding a goal prioritization support component within DHIs are needed.

Interestingly, none of the three priority-prompting DHIs provided guidance on how to decide which health domains receiving a similar rating (eg, a red light) should be prioritized. Furthermore, the three DHIs showed substantial variety in determining or prescribing how long priority goals should stay priorities. In contrast to the once-yearly selection of a priority goal presented in one DHI (J), another DHI (B) prompted users to select one (B1) or two (B2) behavioral goals to work on over a three-month period. The third (F) allowed users to set their own pace, with no apparent limit to the number of domains or behaviors that could be added to their health-to-do-list and no restrictions on when and how often they could add or remove health domains or behaviors from the list. Ideally, prioritization support helps the user not only in setting the right priorities, but also in determining progress towards them over time and deciding when it might be useful to change or take on new priorities [119]. None of the DHIs we found were designed to do this. Just two of three DHIs that prompted prioritization (B and F) explicitly prompted the user to re-engage over time. Only one DHI (D) prompted users to assess progress made towards change goals with the potential to revise their action plans, but it was left unclear how this was achieved. It would be worthwhile for future DHI developers to explore how to optimize training in goal prioritization and management of multiple goals, ideally drawing on research among those who are and are not successful in managing multiple goals and in maintaining behavior change over time [123].

### Limitations

Several limitations are worth noting, both in relation to the review process and the evidence presented. First, although this review used comprehensive search strategies across multiple databases paired with lenient inclusion criteria to ensure that any pertinent literature was reviewed, a substantial number of papers (25/56) were found through forward and backward searches of included publications. This is not surprising, because many included DHIs focused on conducting a health risk assessment, without explicitly noting that this served the purpose of assisting with prioritization of health-related change goals. Nonetheless, it is possible that the review process missed some additional DHIs of this kind. Second, although our review was global, it is interesting to note that, of 19 identified DHIs, eight were developed in the Netherlands and five in the United States. Further, all included DHIs were developed in Western countries. Two excluded DHIs used Traditional Chinese Medicine [124] and Traditional Korean Medicine [125] but did not assess health domains or health-related behavior patterns prior to providing advice. Third, although we found very high inter-rater reliability in relation to our carefully defined inclusion criteria and checked any references that appeared to be ambiguous, we note that, while considerably more expensive, 100% intercoding reliability checks for both abstracts and full papers would have provided greater assurance of inclusion accuracy. Fourth, our review focused on prioritizing health domains and their associated change goals. As a result, it excluded DHIs focused on supporting prioritization of behaviors (singly or together) within domains, where there are multiple options, and of theoretically grounded advice on selecting specific change techniques where multiple possibilities exist. See Tighe et al [126] for a review of such DHIs.

Unfortunately, DHI reports did not always contain sufficient details on the characteristics examined in this review. This hinders empirical comparisons of DHIs and their effectiveness and highlights the importance of detailed intervention design and content reporting in the scientific literature [107,108]. Future DHI evaluation reports should provide clearer details of the dose and duration of intervention components within user sessions and across user engagement (over multiple sessions). For example, how often are DHIs used and which intervention components are available if users revisit the DHI?

Another challenge to the comparison of DHIs and assessment of their effectiveness is that prioritization support results in different individuals pursuing different change goals within the same DHI, meaning there is no common outcome metric. Researchers should not shy away from this challenge. For example, the creators of the Gabby DHI (F) attempted to mitigate complexity associated with multiple outcomes by including summative and percentual outcomes (ie, total number of risks reported at time 1 resolved at time 2; percentage of risks reported at time 1 resolved at time 2) both overall and within specific risk categories. More, such work, is needed to be able to sensibly assess the extent of engagement and outcomes within a common metric that considers the magnitude and likely effort required to make individual changes. For example, quitting smoking requires a large binary shift, but outcomes for an exercise change could be measured using a continuous scale and allow for small changes to be considered successes, making a simple additive outcome measure problematic.

### Implications for DHI Development

These limitations notwithstanding, several implications arise from our findings and the broader literature on goal prioritization.

1. There is considerable potential to develop better goal setting and goal prioritization support in DHI functionality. Most existing DHIs are designed for users who know what domains and associated behaviors they want to prioritize. Prioritization support is needed for the many potential users who have not yet decided.
2. DHIs should include person-based assessments that assist the user to identify what is most important for them given multiple possibilities of different potential benefits (based on theory and empirical data) and their personal needs and preferences. This is likely to optimize DHI effectiveness.
3. DHI development needs to be grounded in an understanding that prioritizing is not, or at least should not be, a one-off activity. This requires DHIs to maintain a relationship with users and support changes in prioritization as needed (eg, once the behavior has been satisfactorily changed and a new one needs choosing).
4. Successful assessment and prioritization should result in a better overview of change options that have the potential to provide substantial health benefits and greater commitment to the prioritized goal, resulting in greater persistence of efforts and eventually greater success [15,25,26,28,104].
5. Improved guidance on the reporting of DHI functionality and capacity is critical to future effectiveness improvement. At present, the standard of reporting means that there is a considerable degree of guesswork involved in describing what any particular DHI offers users [107].
6. Intervention development is likely to be enhanced using co-design and design planning procedures such as those used in intervention mapping [101,106,107].

### Conclusions

Prioritization of goals within the context of multiple goal management has been found to promote goal achievement [25-28]. We found just 19 DHIs that combined health and health behavior assessment with some form of goal prioritization support. All of these could be improved but collectively they identify a range of useful approaches to prompting behavioral assessment, goal setting, and goal prioritization. Providing improved and more comprehensive interventions to scaffold goal setting and prioritization should be a priority for digital intervention designers aiming to promote behavior change among users. The lack of rigorous evaluations means it is as yet unclear how such scaffolding may be designed to be most effective. Long-term follow-up using RCTs would help clarify the real-world impact of these interventions on individual and public health.

## Appendix

Multimedia Appendix 1 PRISMA-ScR checklist.

## Abbreviations

DHI: digital health intervention
HRA: health risk assessment
PRISMA-ScR: Preferred Reporting Items for Systematic Reviews and Meta-Analyses extension for Scoping Reviews
RCT: randomized control trial
